# “Revising the DSM-VI”: Global perspectives on power and classification from lived expertise leadership

**DOI:** 10.1371/journal.pmen.0000626

**Published:** 2026-05-19

**Authors:** Matthew Jackman, Parth Sharma, Agus Sugianto, Anne Meine Pia Van der Schaar, Sasha Hajj-Assaf, Kat McIntosh, Vesper Moore, Angelica Chiketa Mkorongo, Carolina Diaz

**Affiliations:** 1 Centre for Disability Research and Policy, University of Sydney, Sydney, Australia; 2 Sangath, Socorro, Goa, Bhopal, India; 3 Department of Health and Sciences, University of Manchester, Manchester, United Kingdom; 4 Department of Hermeneutical (In)justice and Psychiatric Diagnoses Care Ethics, University of Humanistic Studies, Utrecht, Netherlands; 5 Justice for Mental Health, Beirut, Lebanon; 6 Global Nomads, New York, United States of America; 7 Kiva Centers, Worcester, Massachusetts, United States of America; 8 Pan African Network of Persons with Psychosocial Disabilities, Harare, Zimbabwe; 9 Coalición Neurodivergente Peruana, Lima, Peru; PLOS: Public Library of Science, UNITED KINGDOM OF GREAT BRITAIN AND NORTHERN IRELAND

## Introduction

The American Psychiatric Association’s (APA) Diagnostic and Statistical Manual of Mental Disorders (DSM) is currently undergoing a revision from its fifth edition towards being a “living document,” tentatively titled the *Diagnostic and Scientific Manual* [1] and is strategically shifting away from the term ‘Statistical’. This update by the American Psychiatric Association (APA) purportedly seeks to address social determinants, dimensionality and incorporate the voices of the wider public and those with lived experience in response to long-standing critiques [1]. The DSM operates as a dominant framework, shaping the psychiatric paradigm and consequent diagnostic and treatment practices far beyond the United States of America. As the DSM’s influence expands, this opinion from global lived experience leaders from India, Indonesia, Lebanon, the Netherlands, Peru, Trinidad and Tobago, the USA and Zimbabwe warns that the revision risks reinforcing biomedical dominance without addressing the social, cultural, spiritual, economic, commercial and political realities of both the Global Majority and Minority World. We argue that psychiatric reform requires moving beyond scientifically, philosophically and epistemically limited technical updates and discourse so that language shifts toward a recognition of plural epistemologies and rights-based care that addresses social injustice [2]. Furthermore, it is important to hold space for reform and abolition for new alternative frameworks that are universally humane and reflective of people’s explanatory frameworks of madness and the influential social context. Moreover, Mad Studies and people with lived expertise have long theorised and philosophised explanatory frameworks outside the biomedical and diagnostic model, offering relational, social, and meaning-centred understandings of distress and madness, grounded in lived experience [3].

## Historical context and the expansion of pathology

The history of the DSM is characterized by a steady expansion of medical ‘psychiatric’ authority. From its inception in 1952 with 106 diagnoses, the DSM has ballooned to over 300 categories in the current DSM-5-TR [4]. This proliferation often involves the renaming and relocation of outdated labels rather than their removal; thereby maintaining a medical gaze on social identities, and the matrix of privilege and oppression as indicated by histories of racism, sexism, classism, ableism, sanism and homophobia forming part of formal diagnoses. Crucially, psychiatric diagnoses have historically intersected with structural violence and social control [5,6]. For instance, in the United States, schizophrenia diagnoses were disproportionately applied to Black men during the civil rights era to pathologize resistance and anger [4].

## Core critiques of the DSM-VI framework

Evidence suggests that approximately 60% of DSM-5-TR task force members received personal payments from pharmaceutical companies, totalling more than $14.2 million, highlighting ongoing concerns about conflicts of interest and potential industry influence on diagnostic development. These concerns intersect with critiques that many psychiatric classifications lack robust scientific validity while contributing to the pathologisation of human distress and experiences of madness [2].

A major concern involves the co-option of resistance ‘Mad’ movements [1,2,6]. Although the APA now invites “lived experience” perspectives, in reality, any contributions do not reflect the heterogeneity of the Global Majority, particularly those facing poverty, racism, incarceration, colonial legacies and/or displacement [7,8]. In the Netherlands, it is evident that radical community knowledge is being silenced, as those who most fundamentally challenge the diagnostic paradigm are the least likely to be included in institutional reform [7,8].

While DSM-5 did consider a more neurobiological approach, it was not adopted, reflecting the consensus that, with the recent exception of Alzheimer’s disease, no biomarkers have had the needed specificity and sensitivity for use in routine psychiatric diagnosis and that well-defined pathophysiological mechanisms for psychiatric disorders are limited [9]. There are no brain or blood tests for any psychiatric diagnosis.

## Global perspectives: structural power and contextual mismatch

Synthesizing global perspectives reveals that the DSM often acts as a mandatory gatekeeper to social legitimacy and resources [8]. This gatekeeping is intensified in the fragile health systems of both Peru and Lebanon, where “brief consultations” of only fifteen minutes lead to rapid-fire diagnoses and medication as the default response [10,11].

The prevalence of Western frameworks results in epistemic injustice; local cultural understandings are obscured or pathologized. In Trinidad and Tobago, the DSM lacks the capacity to hold Caribbean frameworks, as the psychological harm of post-colonial identity and Eurocentric imposition is scarcely even acknowledged. In Indonesia, diagnostic labels are often interpreted as fixed, shameful identities rather than clinical tools, leading to social exclusion that is frequently more burdensome than the distress and/or madness itself [11,12]. Finally, in Zimbabwe and Peru, indigenous traditions that view “madness” through spiritual and/or relational lenses are rarely recognized as legitimate knowledge within formal, biomedical mental health systems [10–12].

Amid the ongoing wars and crises occurring within Lebanon and surrounding regions, the DSM creates a classification of who conforms and who does not, which fails to capture the wider structural context of lived realities [13]. The pathologization of rational responses to injustice reframes systemic violence as individual dysfunction [13]. This is evident in global contexts, whether it is war in Lebanon or farmers’ suicide, homelessness and poverty in India [14].

## Alternatives and future directions

Despite the dominance of the DSM, community-rooted and rights-based alternatives are flourishing globally. Voluntary Sanctuaries in the USA provide peer-run respites, while Soteria Houses offer non-carceral support rooted in self-governance and the legal right to community-based care [15]. Through Communal Witnessing, faith communities and spiritual networks in Indonesia and the Caribbean provide meaning-making and collective support that underfunded psychiatric systems cannot offer [11,12]. In terms of artistic and cultural resistance, traditions like rapso and calypso in the Caribbean allow for the collective processing of grief and rage, shifting the focus from individual trauma to social survival [16]. Survivorled advocacy groups like Justice for Mental Health (JMH) in Lebanon advocate for dignified support that is voluntary and provided on the individual’s own terms [17].

Alternative frameworks, such as the Power Threat Meaning Framework, offer ways to understand distress and/or madness in its social and political context rather than through biomedical checklists. These cross-cultural models are humane frameworks which emphasize that recovery begins with dignity, security, and the restoration of social roles rather than the assignment of a permanent label.

## Conclusion

The DSM-VI revision represents a pivotal moment to reflect on the structural limitations of psychiatric classification. While the APA seeks a more “scientific” manual, global perspectives suggest that the current paradigm often reinforces stigma, pathologizes social suffering, and marginalizes indigenous and lived-experience knowledge [1,2,6]. Lived expertise informed reform demands more than updated criteria; it necessitates an honest confrontation with the power structures that diagnostic manuals uphold [13]. For the Global Majority, the future of mental health lies in pluralistic, community-grounded approaches that prioritize dignity and collective care over a universal checklist. There are examples such as the Power Threat Meaning Framework which seeks to understand distress and/or madness from a non-pathologizing and humane perspective. We need radical reform, and alternative worldviews on distress and madness, to care and heal in ways that don’t further medicalise humanity and traumatise unintentionally.
